# Walking the Tightrope: Counterproductive Work Behavior as Compensation for Citizenship Demands

**DOI:** 10.3389/fpsyg.2016.01530

**Published:** 2016-10-03

**Authors:** Andromachi Spanouli, Joeri Hofmans

**Affiliations:** Work and Organizational Psychology, Department of Psychology, Faculty of Psychology and Educational Sciences, Vrije Universiteit BrusselBrussels, Belgium

**Keywords:** organizational citizenship behaviors (OCB), counterproductive work behavior (CWB)

## Abstract

Amidst a struggling economy, organizations are ruled by the survival of the fittest paradigm but it is the employees who tend to pay the price, with increased demands which, oftentimes, fall outside their job scope. In the present paper, we examined whether pressuring people to engage in such organizational citizenship behaviors (OCB) might in fact backfire and lead to increased Counterproductive Work Behavior (CWB) because of compensatory mechanisms. We propose a typology of OCB that distinguishes between discretionary and elicited OCB, hypothesizing that elicited but not discretionary OCB, positively relates to CWB. By doing so, we wanted to examine if such a distinction can explain conflicting past results concerning the within-person OCB–CWB link, and to test whether increased citizenship demands can have an adverse effect for the organization. Our hypothesis was tested by asking 29 employees twice a day for 10 consecutive working days to report on the elicited and discretionary OCB and CWB they performed (*N* = 210 responses). Multilevel logistic regression analyses showed that elicited OCB was positively related to CWB, whereas discretionary OCB was not related to CWB. This finding steers theorizing away from the conventional classification of employees as bad apples versus good soldiers, by revealing that CWB can positively relate to OCB as a result of compensatory mechanisms. From a practical point of view, our results imply that employers should be mindful of the unintended consequences that OCB might entail when employees perceive that they are expected to engage in such behaviors.

## Introduction

Traditionally, work and organizational researchers and practitioners alike have strongly focused on enhancing behaviors that are deemed beneficial for organizations, often without questioning whether they are actually effective, and if so, at what cost (Bolino et al., 2004). Organizational citizenship behavior (OCB) undoubtedly belongs to this category of behaviors, representing employee behavior that lies outside the spectrum of tasks included in the job description and that promotes organizational functioning (Lee and Allen, 2002). Although OCB has long been considered to have only positive consequences both for the organization and the employee, cracks are starting to appear in its exclusively constructive image (Bolino and Turnley, 2005; Bolino et al., 2010). This awareness is particularly important as research indicates that today’s employees not only experience greater in-role work requirements than employees in former times (Feldman, 2002; Brett and Stroh, 2003), but that they also often experience pressures to engage in extra-role behaviors such as OCBs (Bolino et al., 2010). As a result of the recent economic crisis, this trend has only accelerated with employees facing an increasing amount of stressors, such as heightened job demands and role conflict (Giorgi et al., 2015a). The result of these increases in work stressors is that both the mental and general health of the workforce is affected, as shown by increased rates of depression, anxiety, cardiovascular diseases, and respiratory health problems (Mucci et al., 2016). In reference to OCB, research has shown that pressuring employees to engage in OCB can seriously endanger employee wellbeing as it might, for example, lead to increased levels of work-family conflict, work-leisure conflict and job stress (Bolino and Turnley, 2005; Bolino et al., 2010). In the present paper, we take research on OCB one step further by examining the relationship between OCB and counterproductive work behavior (CWB), which consists of employee acts that harm the organization and/or its members (Spector and Fox, 2002), In doing so, we put forward the idea that pressuring people to engage in OCB might not only be detrimental for employee wellbeing, but also for the organization itself, as elicited OCB might backfire and lead to increased CWB because of compensatory mechanisms.

On the basis of their construct definitions, OCB and CWB have traditionally been considered semantic opposites. This conceptualization has led to the formulation of the so-called continuum hypothesis (Heckert and Heckert, 2002) which states that OCB and CWB are two extremes of the same continuum, representing behaviors and acts that help and harm the organization, respectively (Dalal et al., 2009). The continuum hypothesis has been the prevailing proposition to date, with a great deal of empirical evidence pointing toward a strong negative relationship at the between-person level (e.g., Bennett and Stamper, 2001; Sackett, 2002), suggesting a clear differentiation of employees in two groups: those who generally perform OCB (and not CWB) and those who generally perform CWB (and not OCB). However, despite this initial apparent support, the meta-analysis of Dalal (2005) demonstrated that methodological artifacts distorted the OCB–CWB relationship, and that when these artifacts are accounted for, the OCB–CWB relationship is actually modestly negative. Moreover, recent studies at the within-person level also reported weak negative, and in some cases even positive OCB–CWB relationships (Dalal et al., 2009; Ilies et al., 2013).

With the present study, we aim to provide an explanation for these mixed findings by studying the circumstances under which the within-person OCB–CWB relationship may differ. Toward that end, we introduce a distinction between discretionary and elicited OCB. Discretionary OCB concerns behaviors that employees choose to perform out of free will, while elicited OCB refers to behaviors that employees engage in as a result of a perceived obligation. Elicited OCB is in line with Spector and Fox’s (2010) concept of demand-elicited OCB, which pertains to citizenship behaviors that are performed due to demands that elicit such behavior; thus introducing an element of obligation to the traditional OCB construct while stripping it from its voluntary components. Moreover, it bears similarities with Vigoda-Gadot’s (2006) compulsory citizenship behavior and Bolino et al.’s (2010) citizenship pressure. Although we are not the first to introduce the idea of elicited OCB, previous studies were either theoretical (Vigoda-Gadot, 2006; Spector and Fox, 2010), tested elicited OCB in relation to different antecedents or outcomes (Bolino et al., 2010), or focused on the between-person level (Vigoda-Gadot, 2007). This study takes the current state of affairs a step further by empirically testing the effect of both OCB types on CWB at the within-person level, thereby trying to explain how the same individual may engage in both forms of behavior. To this end, we draw from different theoretical frameworks that provide the groundwork for understanding these relationships. But first, we zoom in on the main constructs of this study: OCB and CWB.

### OCB and CWB

Organizational citizenship behavior has received extensive research attention since the seminal work by Smith et al. (1983) and Organ (1988). In its early conceptualizations, OCB was defined as *“individual behavior that is discretionary, not directly or explicitly recognized by the formal reward system, and that in the aggregate promotes the effective functioning of the organization”* (Organ, 1988, p. 4). Three distinct components are clear in this initial definition: (1) the lack of compensation for such behaviors, (2) their discretional nature, and (3) the beneficial effect that they have for the organization.

Much has changed since this initial conceptualization as critics have raised points concerning each of these components. In terms of (1) the lack of compensation, studies have revealed that managers actually do take into consideration citizenship behaviors when rating their subordinates performance (e.g., Podsakoff et al., 2000, 2009), a finding which led to the exclusion of this component from current definitions. Concerning (2) the discretional nature of such behaviors, although there have been scholars who challenged this assumption, these studies often form a parallel stream of literature, frequently coupled with distinct constructs and conceptualizations, thus treating non-discretionary OCB as a separate phenomenon (Vigoda-Gadot, 2006, 2007; Bolino et al., 2010). Hence, although there seems to be consent that OCBs are often not voluntary, the conceptualization and often the corresponding measurement in the majority of the existing studies do not allow for such a differentiation. To account for this issue, we introduce the distinction between elicited and discretionary motives as two distinct drivers for the same types of citizenship behaviors. Regarding (3) the beneficial effect OCBs have for the organization, meta-analytic studies were able to confirm positive relationships of OCB with organizational outcomes such as performance, reduced costs and customer satisfaction (Nielsen et al., 2009; Podsakoff et al., 2009). However, there have also been, if only but a few, studies that have warned about the possible negative consequences that OCB may bare, both for organizations and for their enactors (for an overview, see Bolino et al., 2013). In this paper, we take stand with this account by introducing CWB as a potential outcome of OCB.

Counterproductive work behavior is an umbrella term that can encompass such distinct behaviors as production deviance, abuse, and withdrawal (Spector et al., 2006). Though its components are so distant from each other, they all share the principle of being considered harmful for an organization (Sackett, 2002; for a different approach, see Krischer et al., 2010). CWB has traditionally been studied at the between-person level, with its main predictors being personal or situational characteristics (Penney and Spector, 2005; Iliescu et al., 2015). Although the behaviors that comprise this construct are as equally distinct as the reasons why one would engage in such behaviors, only a few studies have focused on the variation of CWB within individuals (Dalal et al., 2009; Ilies et al., 2013; Debusscher et al., 2016). In the present study, we examine how CWB can fluctuate within the individual in relation to OCB. In order to do so, we proceed with presenting some theoretical approaches that explain how elicited and discretionary OCB and CWB can be related at the within-person level.

### Theoretical Approaches

One size does not fit all individual behaviors; therefore, we chose to present three theoretical frameworks which complement each other in regards to the predictions they offer on how OCB and CWB might relate at the within person level: equity theory (Adams, 1965), conservation of resources (COR) theory (Hobfoll, 1989), and self-determination theory (SDT) (Deci and Ryan, 1985) (for other theoretical accounts, see Spector and Fox, 2010 and Klotz and Bolino, 2013).

### Equity Theory and Fairness Model

Equity theory (Adams, 1965) focuses on individuals’ perception of fairness. More specifically, it maintains that employees’ equity perceptions are determined by comparing whether the ratio of their own contributions (input) and the benefits they receive in response (output), matches that of a comparison other. The fairness model (Carrell and Dittrich, 1978) extends equity theory by establishing that an external comparison entity is not a prerequisite and that an internally derived standard can also be used for such comparison. According to both models, whenever individuals find themselves in an inequitable situation, they take actions directed at restoring equity levels.

Drawing on equity theory and the fairness model, we posit that elicited OCB is considered as an input by its enactor as it consists of positive investments that one is obliged to fulfill for the benefit of the organization. As a result of this obligation, the enactor feels entitled to receive a fair output in return. If employees perceive that their organization fails to provide something in return and that their inputs outweigh their outcomes, they can experience distress since they are not free to withhold elicited OCB as a strategy to balance their inputs and outputs. Moreover, since OCB typically lies outside their contractual obligations, employees may perceive the pressure to engage in such behaviors as unfair (Vigoda-Gadot, 2006). Therefore, in an attempt to restore their equity levels employees may seek restitution by engaging in CWB (Beauregard, 2014).

Performances of discretionary OCB, on the other hand, although they contribute *to* the benefit of the organization, are not necessarily performed *for* the organization. We posit that individuals who voluntarily choose to engage in such behaviors do not consider them as inputs and thus do not expect a certain organizational output in return. Hence, we argue that discretionary OCB has no impact on its agents’ input/output ratio and by extension neither on their equity perceptions. Conclusively, we expect that discretionary OCB will not be related to manifestations of CWB.

### Conservation of Resources Theory

Conservation of resources theory maintains that individuals go to great lengths to preserve or increase their existing pool of resources, to the extent that, when individuals face an actual, or even a potential resource loss, they experience psychological stress (Hobfoll, 1989, 2002). What constitutes resources depends on what people value or what people use in order to acquire what they value (Wells et al., 1999), which ultimately falls into four main categories: objects (e.g., house), personal characteristics (e.g., self-esteem), conditions (e.g., marriage), and energies (e.g., time).

Energy resources fluctuate greatly since they are the resources that individuals tend to invest in order to acquire more resources (Hobfoll, 1989). In a work context, employees are expected to invest both time and energy to fulfill their job requirements. However, as OCB falls outside their formally assigned tasks, employees may experience the obligation to engage in OCB as a superfluous constraint, which will potentially deplete their energy resources. When faced with such constraints, individuals try to restrain their (potential) losses and one such strategy is by engaging in CWB (Fox and Spector, 1999; Krischer et al., 2010).

On the contrary, when employees themselves choose whether or not and when they engage in OCB, this shall not pose a threat to their pool of resources since they will also have the choice to cease this behavior. What is more, employees may even regard some forms of OCB (i.e., helping behavior) as a resource investment (Hobfoll, 1989), and therefore their engagement in discretionary OCB will not relate with acts of CWB.

### Self-determination Theory

Self-determination theory holds that motivation varies not only in regards to its quantity but also to its quality (Deci and Ryan, 2008). The theory discerns among six types of motivation (for more info, see Deci and Ryan, 1985; Ryan and Deci, 2002) which fall under three main categories: amotivation, controlled motivation, and autonomous motivation. As its name gives away, amotivation refers to lack of intention to act or, otherwise stated, lack of motivation. Controlled motivation concerns motivation that is externally regulated, whereas autonomous motivation encompasses self-motivated behaviors (Ryan and Deci, 2000). Although the different types of motivation are clearly distinguishable among each other, they are not static (Bidee et al., 2016). According to SDT, individuals experience motivation in relation to the satisfaction of three psychological needs: the need for competence, the need for relatedness and the need for autonomy (Deci and Ryan, 2008; Vandercammen et al., 2014). Satisfaction of these needs can thus transform a controlled motivated behavior to autonomous, with studies showing that one key element for such a transformation is the satisfaction of the need for autonomy (Gagné and Deci, 2005).

In relation to OCB, although the performance of two such acts may seem identical, the motivation that drives these behaviors can lead to different outcomes. In particular, when employees feel obliged to engage in OCB, they are less likely to experience autonomous motivation, since their behavior has an external locus of control, which can hinder an individual’s need for autonomy. As a result, employees may choose to engage in CWB in order to balance these demands.

Conversely, when employees choose themselves to engage in OCB, they are more likely to do so as a result of autonomous motivation. They may choose to perform OCB due to a genuine interest or due to the values they attach to such behaviors. In any case, when they choose such an activity voluntarily, their needs satisfaction is not under threat and therefore their engagement in discretionary OCB would not result in CWB.

Combining the insights of equity theory, COR theory and SDT, we expect that elicited OCB will be positively related to CWB whereas discretionary OCB will be unrelated to CWB:

H1: *Elicited OCB relates positively to CWB at the within-person level*.
H2: *Discretionary OCB is unrelated to CWB at the within-person level*.

## Materials and Methods

### Participants and Procedure

Twenty-nine employees, of whom 17 were females, participated in an experience sampling study which took place in Belgium. Respondents were on average 30 years old (*SD* = 9.70) and their organizational tenure varied between 0 and 24 years (*SD* = 5.92). They all held full time jobs, and were employed in a variety of sectors including, among others, healthcare, banking, education, justice, and journalism. The majority of them (51.7%) held a Master’s degree.

As our hypotheses pertain to within-person fluctuations in OCB and CWB, traditional cross-sectional designs do not suffice since they do not allow the capturing of such day-to-day fluctuations. Instead, we used an experience sampling design, which is currently considered to be the best available option when the goal is to capture short-term changes in real time and in the person’s actual environment (Fisher and To, 2012). In our experience sampling study, participants were given a smartphone for 10 consecutive working days on which they received a survey at midday and another one at the end of their working day. At both points in time, they completed the same survey, measuring OCB, CWB and a number of discrete emotions (note that the emotions are not used in the study). The study was approved by the Human Research Ethics Committee of the Vrije Universiteit Brussel (reference number ECHW_049). All respondents signed an informed consent form prior to participating, and in this informed consent it was indicated that they had the choice to withdraw from the study at any point. Out of a maximum 580 observations (29 participants × 10 days × 2 measurement moments), 210 observations were recorded, which equals a 36.2% response rate.

### Measures

Organizational citizenship behavior and counterproductive work behavior were measured using Dalal et al.’s (2009) scale, which was specifically designed for experience sampling research. Participants were requested to indicate whether they performed the behaviors since that morning (if it was the midday beep) or since the previous beep (if it was the end-of-the-working-day beep). As previous studies have argued in favor of facet dimensionality (Bennett and Robinson, 2000; Dalal et al., 2009), we chose to distinguish behaviors directed toward individuals (OCBI and CWBI) from those targeted at the organization (OCBO and CWBO). Six OCBI and six OCBO items comprised the OCB scale. Example items were “I tried to help a co-worker” and “I went above and beyond what was required for the work task.” The same distinction pertained to CWB, including six CWBI and six CWBO items. Example items were “I spoke poorly about a co-worker to others” and “I worked slower than necessary.” Response options were “yes” or “no.”

The distinction between elicited and discretionary OCB was accomplished with an additional item. When employees indicated that they performed a particular OCB, they received a follow-up question asking whether they performed the behavior either because they felt obliged or because they wanted to themselves, representing elicited and discretionary OCB, respectively.

### Analysis

We first dummy coded the OCB and CWB scale scores because they were heavily skewed with little differentiation in the scale scores besides the absence/presence of OCB and CWB. Subsequently, we tested the relationships between the different types of OCB and CWB. In order to account for the hierarchical structure of our data and because the outcome variables (i.e., CWBI and CWBO) were dichotomous in nature, we tested our hypotheses using multilevel logistic regression in the lme4 package in R.

Two series of multilevel logistic regression models were tested. Elicited OCBI/O and discretionary OCBI/O were separately entered as predictors, while CWBI served as the outcome variable in the first and CWBO in the second series. For each of these models we tested whether the effect of the predictor was fixed or random across participants by comparing the model with a fixed slope to that with a random slope using a χ^2^ difference test. If the random slope model fitted the data significantly better than the fixed slope model, the random slope model was retained; if not, the fixed slope model was the final model (Hox, 2010). Because all hypotheses pertain to the fixed effects, we report only these effects in the “Results” section.

## Results

**Table 1** contains descriptive statistics, intra-class correlation coefficients (ICC) and within-person correlations for all study variables. The ICC’s reveal that a substantial amount of variability in all study variables was situated at the within-person level. Because very little variability was found on the day-level, we proceeded with two-level regression models (Hox, 2010).

**Table 1:** Study means, standard deviations, and within-person correlations.

	*M*	*SD*	*ICC*_Person_	*ICC*_Day_	*ICC*_Moment_	1	2	3	4	5
1. CWBI	0.46	0.50	0.28	0.06	0.66					
2. CWBO	0.54	0.50	0.24	0.00	0.76	-0.06				
3. Discretionary OCBI	0.96	0.20	0.64	0.00	0.36	0.06	-0.00			
4. Discretionary OCBO	0.82	0.39	0.58	0.00	0.42	-0.04	-0.08	0.15^∗^		
5. Elicited OCBI	0.23	0.42	0.34	0.07	0.59	0.18^∗∗^	0.13	-0.05	-0.07	
6. Elicited OCBO	0.32	0.47	0.31	0.07	0.62	0.24^∗∗^	-0.07	0.05	-0.26^∗∗^	0.23^∗∗^

*^∗^*p* < 0.05; ^∗∗^*p* < 0.01.*

Regarding the relationship between elicited OCB and CWB, we found a positive same-target effect of OCBI on CWBI (γ = 1.36; *p* = 0.003), and no same-target effect of OCBO on CWBO (γ = -0.04; *p* = 0.913). For the cross-target effects, we found a positive relationship both between OCBO and CWBI (γ = 1.01; *p* = 0.006), and between OCBI and CWBO (γ = 0.73; *p* = 0.080), with the latter relationship approaching conventional levels of significance. Together, these findings show that, when individuals felt obliged to engage in OCB (either toward the organization or toward co-workers), they were more likely to engage in CWB as well. As a result, our findings generally supported Hypothesis 1.

In line with Hypothesis 2, discretionary OCB was unrelated to CWB. Non-significant effects were found both for same-target (γ = 0.47; *p* = 0.573 for OCBI with CWBI and γ = -0.38; *p* = 0.409 for OCBO with CWBO) and cross-target relationships (γ = -0.23; *p* = 0.614 for OCBO with CWBI and γ = 0.61; *p* = 0.485 for OCBI with CWBO). In other words, when individuals voluntarily engaged in OCB (either toward the organization or toward co-workers), they were equally likely to engage in CWB when they did not voluntarily engage in OCB.

## Discussion

In the present paper, we challenged the idea that OCB and CWB are located at the extreme ends of the same continuum and should therefore be highly negatively correlated (Heckert and Heckert, 2002). Instead, we suggest that the relationship between OCB and CWB can differ depending on the reason why one engages in citizenship behaviors. Our findings generally supported this idea and showed that distinguishing between elicited and discretionary OCB helps to arrive at a better understanding of the OCB–CWB relationship. In particular, when individuals engage in discretionary OCB, they do so, as its name indicates, out of free-will, and therefore such manifestations of discretionary OCB do not relate to manifestations of CWB. On the other hand, when employees have no choice but to engage in OCB because of a perceived obligation, they may seek restitution with behavioral responses such as CWB. Although we also found a weak marginally significant relationship between OCBI and CWBO, these CWBs turned out to be primarily targeted toward other individuals. One reason for this might be that, because employees view the behaviors targeted toward the organization as more closely related to their overall job performance (Dalal et al., 2009), they may perceive that CWBs toward the organization have more severe consequences for their performance evaluations and thus avoid engaging in them.

By showing that elicited but not discretionary OCB related to CWB, our findings provide strong counterevidence for the continuum hypothesis, thereby steering theorizing away from the conventional classification of employees as bad apples versus good soldiers. Moreover, they might explain why recent within-person studies on the relationship between OCB and CWB found mixed results (Dalal et al., 2009; Ilies et al., 2013). From a practical stance, the results suggest that organizations and managers should be mindful of the consequences that increased demands may bring upon, as demands for enhanced OCBs can coincide with unwarranted CWBs. This implication is particularly relevant nowadays, as the boundaries between in-role and extra-role performance seem to be increasingly obscured, especially in economies that were hit by the recent crisis. Finally, our study also complements the findings of recent studies on the effect of increased demands (during for example the economic crisis) by showing that these increased demands take their toll not only on employees (Mucci et al., 2016), but also on organizations.

Despite its strengths, this study also comes with some limitations. Although we used an experience sampling design, which is currently regarded as the “golden standard” when it comes to the tracing of human activity in everyday life (Kahneman et al., 2004), all study variables were self-reported, which can give rise to concerns of common method bias. Yet, the presence of some non-significant relationships suggests that common method bias may not be a major problem in our data (Lindell and Whitney, 2001). Second, it is important to note that the Dalal et al.’s (2009) scale contains semantic opposites in the OCB and CWB scales (e.g., “chose to work rather than to take a break” for the OCB and “took an unnecessary break” for the CWB scale). This could have the implication of inflating the relationship for the same target effects (Dalal, 2005). However, as we found no significant same target relationships for discretionary OCB and CWB and neither for elicited OCB and CWB toward the organization, we are confident that this does not pose a serious issue. We also need to address that severe counterproductive behaviors were not covered by the CWB measure (Dalal et al., 2009). Given our study design, however, it was very unlikely that such severe behaviors would commonly occur during a relatively short period of 10 working days. Third, we cannot rule out reversed causality. It might for example be that the positive relationship between elicited OCB and CWB is the result of employees’ reframing of their OCB’s as obligatory simply to justify their engagement in CWB. Future research remains to resolve this riddle with the use of time lags. Finally, whereas we drew from equity theory (Adams, 1965), COR theory (Hobfoll, 1989) and SDT (Deci and Ryan, 1985) to make the case that elicited but not discretionary OCB would positively relate to CWB, we did not explicitly test the mechanisms proposed by these theories. Thus, although our study revealed that elicited and discretionary OCB related differently to CWB, future research is needed to identify what the mechanisms are underlying these relationships.

Future studies could also examine the role of other motives to engage in OCBs, by expanding our classification with the addition of motives such as impression management, organizational concern (Rioux and Penner, 2001), or reciprocity (Spitzmuller and Van Dyne, 2013). We are also in need of more research focusing on the aftermath of CWBs for the enactors. If we are to assume that employees use CWBs to compensate for increased demands or stressors, research focusing on daily recovery or general indicators of wellbeing, can determine whether this strategy is effective. Finally, as leaders appear to reflect their own feelings of stress to subordinates (Giorgi et al., 2015b), it is interesting to research this dyadic relationship, and examine whether the leaders’ perceived pressure to engage in citizenship behaviors spills over to their subordinates.

In summary, we uphold that the OCB typology presented in this paper may give rise to a fruitful line of research with important potential implications for theory and practice. On a theoretical level, the distinction between discretionary and elicited OCB not only allows to explain mixed findings on the OCB–CWB relationship, but it might also shed light on previous conflicting results considering OCB’s relation with other antecedents and outcomes (e.g., work-family conflict). Our field can benefit from examining traditional concepts through different lenses, as we showed in this study that the generally considered constructive citizenship behaviors can in fact backfire and cause harm to the organization. In the same manner, CWBs, although generally depicted as malevolent acts, have the potential to help employees counterbalance the increased demands they face, and possibly even protect their wellbeing in the long run. Regarding the practical side, being aware of the distinction between elicited and discretionary OCB and its potential consequences for its relationship with CWB might make organizations and managers more reticent of pressuring people to engage in OCB, which in the end might benefit both the employee and the organization.

## Author Contributions

All authors listed, have made substantial, direct and intellectual contribution to the work, and approved it for publication.

## Conflict of Interest Statement

The authors declare that the research was conducted in the absence of any commercial or financial relationships that could be construed as a potential conflict of interest.

The reviewer JP and the handling Editor declared their shared affiliation, and the handling Editor states that the process nevertheless met the standards of a fair and objective review.
